# A genome-wide association study on androstenone levels in pigs reveals a cluster of candidate genes on chromosome 6

**DOI:** 10.1186/1471-2156-11-42

**Published:** 2010-05-20

**Authors:** Naomi Duijvesteijn, Egbert F Knol, Jan WM Merks, Richard PMA Crooijmans, Martien AM Groenen, Henk Bovenhuis, Barbara Harlizius

**Affiliations:** 1 IPG, Institute for Pig Genetics B.V., PO Box 43, 6640AA, Beuningen, the Netherlands; 2 ABG, Animal Breeding and Genomics Centre, Wageningen University, PO Box 338, 67001 AH Wageningen, the Netherlands

## Abstract

**Background:**

In many countries, male piglets are castrated shortly after birth because a proportion of un-castrated male pigs produce meat with an unpleasant flavour and odour. Main compounds of boar taint are androstenone and skatole. The aim of this high-density genome-wide association study was to identify single nucleotide polymorphisms (SNPs) associated with androstenone levels in a commercial sire line of pigs. The identification of major genetic effects causing boar taint would accelerate the reduction of boar taint through breeding to finally eliminate the need for castration.

**Results:**

The Illumina Porcine 60K+SNP Beadchip was genotyped on 987 pigs divergent for androstenone concentration from a commercial Duroc-based sire line. The association analysis with 47,897 SNPs revealed that androstenone levels in fat tissue were significantly affected by 37 SNPs on pig chromosomes SSC1 and SSC6. Among them, the 5 most significant SNPs explained together 13.7% of the genetic variance in androstenone. On SSC6, a larger region of 10 Mb was shown to be associated with androstenone covering several candidate genes potentially involved in the synthesis and metabolism of androgens. Besides known candidate genes, such as cytochrome P450 A19 (*CYP2A19*), sulfotransferases *SULT2A1*, and *SULT2B1*, also new members of the cytochrome P450 *CYP2 *gene subfamilies and of the hydroxysteroid-dehydrogenases (*HSD17B14*) were found. In addition, the gene encoding the ß-chain of the luteinizing hormone (*LHB*) which induces steroid synthesis in the Leydig cells of the testis at onset of puberty maps to this area on SSC6. Interestingly, the gene encoding the α-chain of LH is also located in one of the highly significant areas on SSC1.

**Conclusions:**

This study reveals several areas of the genome at high resolution responsible for variation of androstenone levels in intact boars. Major genetic factors on SSC1 and SSC6 showing moderate to large effects on androstenone concentration were identified in this commercial breeding line of pigs. Known and new candidate genes cluster especially on SSC6. For one of the most significant SNP variants, the difference in the proportion of animals surpassing the threshold of consumer acceptance between the two homozygous genotypes was as much as 15.6%.

## Background

In many countries, male piglets are castrated shortly after birth to prevent boar taint, which is an urine-like, unpleasant flavour and odour released at cooking or heating of pork [1]. However, recent discussions on the pain associated with castration of the piglets early in life have led to a ban on castration without anaesthesia in some countries. In addition, studies have shown that un-castrated males grow faster and have an improved feed efficiency due to reduced fat deposition [2-4]. In future, if un-castrated males will be finished, boar taint needs to be prevented. Two of the major components related to the boar taint are androstenone and skatole [5-7]. Androstenone (5α-androst-16-en-3-one) is a male sex pheromone produced by the testes and stored in adipose tissue causing a perspiration-like odour [8,9]. Androstenone precursors are also transported to the salivary glands which are capable to produce high levels of androstenone during sexual excitement [10,11]. Skatole possesses strong faecal odour and is produced by the bacterial breakdown of the amino-acid tryptophane in the lower gut [12]. Skatole then diffuses into fat tissue.

There is considerable variation for androstenone and skatole between and within lines of pigs. Especially androstenone has high heritability estimates ranging from 0.25 to 0.88 [13,14]. Somewhat lower heritabilities have been reported for skatole, between 0.19 and 0.55 [15,16]. Two linkage studies using microsatellite markers have identified several QTL regions for androstenone and skatole in experimental crosses with 485 and 187 F2 animals, respectively [17,18] pointing towards several areas in the genome affecting these traits. Also, single candidate genes involved in androstenone synthesis and metabolism of androstenone and skatole have been analyzed at the level of RNA and protein expression and in single SNP association studies (reviewed by Robic et al., 2008 [19]). However, no conclusive results showing functional mutations affecting androstenone and skatole levels in fat tissue have been described until now. Recently, large-scale microarray expression studies have reported hundreds of differentially expressed genes which might be involved in synthesis and degradation of androstenone and skatole in testis and liver [20,21]. Subsequent analysis of SNPs in 121 differentially expressed genes identified 10 genes associated with one of the two traits [22]. Recently, Markljung et al. (2008) [23] reported 2 QTL for androstenone in 139 animals from a cross between Hamphsire and Landrace animals. Although these studies are of limited size and resolution, they indicate that several genetic factors seem to be involved in determining the levels of these boar taint compounds.

Recently, the first high-density 60K porcine SNP array has been developed [24] that offers a much higher resolution. A genome-wide association study (GWAS) was initiated using the SNP array to identify the chromosomal regions and specific SNPs influencing boar taint levels in a commercial breeding population. However, mean skatole levels (75 ng/g fat) in this population were far below the threshold accepted by consumers of 250 ng/g fat [25]. To reduce genotyping costs, a selective genotyping strategy for androstenone was applied. In this study, we present the results of a GWAS in pigs by genotyping 987 un-castrated male pigs from a commercial breeding population with large phenotypic variability for androstenone levels in fat, using the 60K (64,232) SNP array. The GWA resulted in an increased resolution compared to previous linkage studies. A large cluster of candidate genes within a 10 Mb region on SSC6 was identified. In addition, three new areas on SSC1 were detected that affect androstenone levels in this breeding line.

## Methods

### Animals and phenotypes

This experiment was conducted strictly in line with the regulations of the Dutch law on the protection of animals. Phenotypic measurements on androstenone were obtained from 1,663 boars slaughtered at a mean hot carcass weight of 95.71 kg. All the boars were purebred animals from a composite Duroc sire-line. Boar taint compounds were measured using fat samples from the neck collected from the left carcass side. The samples were stored under vacuum at -20°C. For androstenone, a fat extraction was done on the fat samples as described by Tuomola et al., 1997 [26]. Thereafter, androstenone concentrations in liquid fat were estimated by time-resolved fluoro-immunoassay [26] at the Hormone laboratory, Oslo.

Androstenone was not normally distributed and therefore log-transformed (ln-androstenone). The log transformed androstenone values were analysed using the following statistical model using ASREML [27]:

Where y = ln-androstenone; hcw = effect of hot carcass weight as covariate; age = effect of age at slaughter as covariate; fat= effect of fat depth at slaughter as covariate; batch= the random effect of the i^th ^batch, pen = the random effect of the j^th ^pen within the i^th ^batch; litter = the random effect of the k^th ^litter within the i^th ^batch; a = additive genetic effect of the l^th ^animal; e = residual effect. Systematic environmental effects were estimated using the full dataset (N = 1663). In the genome-wide association study, androstenone levels adjusted for systematic environmental effects were used and these were calculated as:

### Selective genotyping

A simulation study was performed in order to select about 1000 animals from 1663 candidates for genotyping in an optimal way using the existing pedigree [28]. Ten markers and 1 QTL were simulated on 1 chromosome and also 1 chromosome was simulated without a QTL for determining the false-positive rate for a given threshold. Four alternatives for selecting 1000 individuals to be genotyped out of 1663 candidates were compared: 1. random, 2. selecting large half-sib families, 3. selecting high and low phenotypes, 4. selecting high and low phenotypes within full sib families. ANOVA was used to analyze each marker and determine the F-statistic. Selection of high and low phenotypes within full sibs showed the highest power (results not shown). Applying the selection of high and low phenotypes (within full sib families) to our data set consisting of 1663 pigs resulted in 987 pigs selected for genotyping. These pigs originated from 57 sires and 212 dams. Among them, 45 sires and 11 dams were available for genotyping.

### Genotyping and quality control

Genotyping was performed using the PorcineSNP60 Beadchip of Illumina (San Diego, CA, USA) [24]. A total of 1043 samples (including sires and dams) were genotyped for 64,232 SNPs at Service XS (Leiden, The Netherlands) and data quality was evaluated. The average call rate for all samples was 98.4% ± 3.4. A total of 63 animals were removed due to pedigree errors (<99% correct genotypes). After quality control, 943 animals were available for the GWAS with 106 singletons and 313 divergent full sib pairs (2 or more full sibs). For the SNPs, a threshold of 30 pedigree errors or more was applied and 190 SNPs were removed. In addition, 10,210 SNPs were removed because of low quality score (GenCall score <0.7). A minor allele frequency of 0.01 was applied removing another 4,925 SNPs of which 980 were monomorphic. In total, 47,897 SNPs remained for the GWAS.

### Genome-wide association analysis

Corrected log-transformed androstenone was analyzed as a quantitative trait under an additive model using the QFAM module of PLINK [29]. The more stringent within-sib-ship test within QFAM was performed which is robust for population stratification compared to the total-sib-ship test. Nominal scores were permuted to obtain an empirical p-value while maintaining familial correlation between genotype and phenotype. The permutation procedure employed by QFAM corrects for relatedness within families and was performed 1,000,000 times. Genomic control was used to correct for score inflation introduced by relatedness between family units (sib ships) [30]. False-discovery rate (FDR) was applied to correct for multiple-testing. The R package q-value [31] was used to calculate a FDR-based q value to measure the statistical significance at the genome-wide level for association studies. The cut-off of significant association at the whole genome level was set at q-value ≤ 0.05. The total variance explained by a SNP was calculated using ASReml version 2.0, [27]. For ASReml the full model (as described earlier and including the polygenic effect) was used for the animals genotyped including the SNP as a random effect.

The fraction of the phenotypic variance explained by the .

Linkage disequilibrium (LD) between SNPs was quantified as r² on all the animals of the GWA study using Haploview (V4.2; [32]) and the LD block was defined by the criteria of Gabriel et al. (2002) [33].

### Identification of candidate genes

Porcine transcripts and annotation were downloaded from the porcine Ensembl data base (build9) and aligned with the human RefSeq mRNA sequences using BLAT [34]. The human-porcine comparative map was calculated based on the orthologous human-porcine transcripts and for the syntenic regions annotations were downloaded from the NCBI database (build37). Additional candidate genes present in human but not identified in the BLAT search against the human transcriptome were mapped to SSC6 performing a BLAST alignment with the porcine cDNA (*SULT2A1*) or the human homolog (*SULT2B1*, *HSD17B14*) against the porcine genome sequence (build9).

## Results

**Figure 1:**
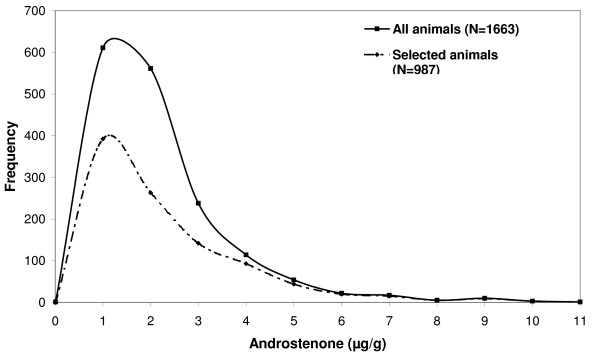
**Distribution of androstenone for the full dataset (N = 1663) and after selective genotyping was applied (N = 987)**.

The descriptive statistics of the phenotypic measurements of the boars used for the GWAS are given in Table 1. Animals were slaughtered at a mean age of 179.80 d with an average carcass weight of 95.7 kg. The average androstenone level was 1.88 μg/g melted fat, and the average skatole and indole levels were 91.11 ng/g and 54.15 ng/g, respectively. The change of the distribution of androstenone concentrations after selection of divergent sib pairs is shown in Figure 1 indicating that not only extreme androstenone levels are represented.

**Table 1:** Descriptive statistics for traits measured.

Trait	N	mean	SD	min	max
*Boar taint compounds*					
Androstenone (μg/g)	943	1.88	1.67	0.07	10.10
Skatole (ng/g)	942	91.11	97.48	6.00	928.00
Indole (ng/g)	942	54.15	64.79	8.00	678.00
Ln-androstenone	943	0.25	0.91	-2.66	2.31
					
*Finishing traits*					
Hot carcass weight	943	95.71	10.95	67.60	136.20
Fat depth at slaughter	943	14.96	2.93	7.60	27.60
Age at slaughter	943	179.80	9.26	152.00	247.00

Mean, standard deviation (SD), minimum (min) and maximum (max) values are presented for all the phenotypes included in the association study (N).

**Figure 2:**
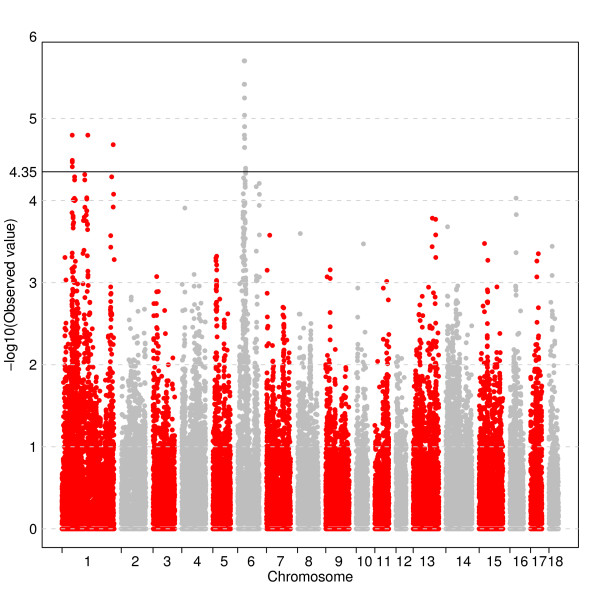
**Association between ln-androstenone and 40,525 mapped SNPs across 18 autosomes using an additive model**. Each dot represents one SNP. On the y-axis are -log10 (p-values), and on the x-axis are the physical positions of the SNPs by chromosome. Cut-off value is 4.35 which equals a FDR q-value ≤ 0.05.

The GWA analysis using the threshold for FDR of q ≤ 0.05 showed that 37 SNPs were genome-wide significantly associated with log-androstenone (Figure 2 and additional file 1). Among them, thirty-five SNPs are located in regions with multiple significant SNPs. Three regions were identified on SSC1, and one larger region on SSC6. The region between 36.9 Mb and 44.9 Mb on SSC6 encompasses a large cluster of 31 significant SNPs. A single SNP analysis of the most significant SNPs on SSC1 and SSC6 using a mixed model and including a polygenic effect thereby correcting for other genetic factors affecting androstenone (background genome) is shown in additional file 1. The fraction of the phenotypic variance explained by a single SNP varies between 1.5% and 5.8%.

**Figure 3:**
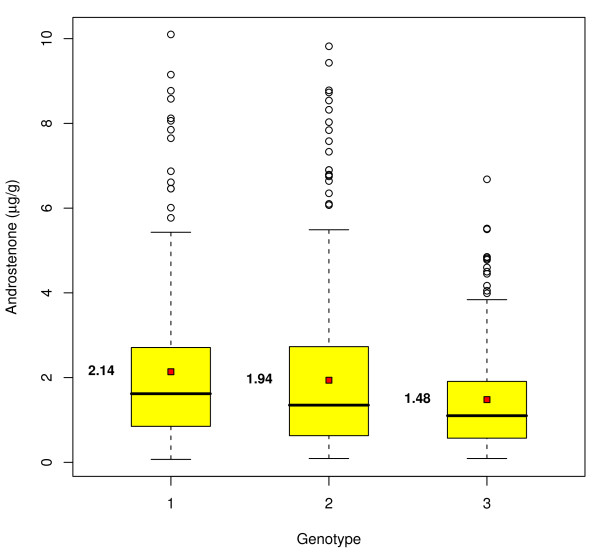
**Box plots of the distribution of the untransformed androstenone concentrations for the SNP MARC0049189 (nr 15)**. The mean is given in bold.

Figure 3 shows the means for the untransformed androstenone levels of the three genotypes of SNP nr 15 on SSC6. There is a difference of 0.66 μg/g between the mean level of the two homozygous genotypes. Correction for systematic environmental effects hardly affects the differences between the genotypes (data not shown). Moreover, among the animals homozygous for the allele associated with high androstenone levels, 39.6% of the animals surpass the threshold of consumer acceptance (2 μg/g). This proportion is markedly reduced by 15.6% in the homozygous low genotypes (24.0% above 2 μg/g). A more detailed view of SSC6 is shown in additional file 2. The high density of genes presently annotated in EnSembl on SSC6 (n = 351, additional file 3) is even more pronounced in the area of interest with a total of 24 genes between 36,9 Mb and 40 Mb. The homologous region in human on HSA 19q13 between 50 Mb and 52,2 Mb is also very gene-rich with a total of 139 genes and 255 transcripts being annotated until now.

**Figure 4:**
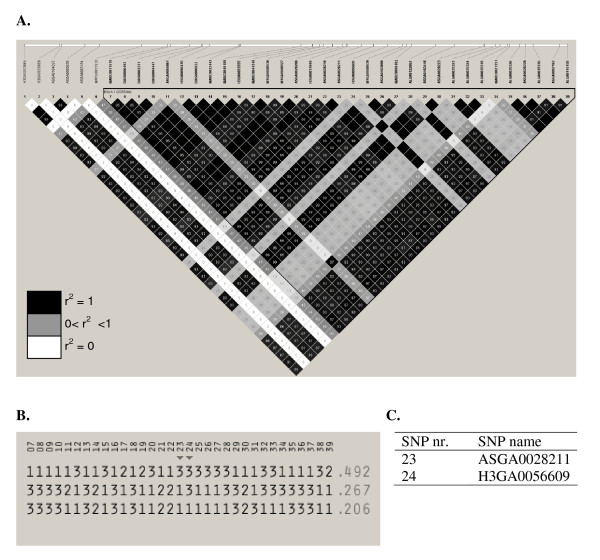
**Linkage disequilibrium plot for the region between 36.9 Mb and 39.7 Mb on SSC6**. All 31 significant SNPs (p ≤ 0.05 after FDR) and intervening SNPs for all animals (N = 943) are shown (**A**). The values in the boxes are pair wise SNP correlations (r²) and the box colour reflects the degree of correlation. **B **Haplotypes with all SNPs from the LD block are shown. Each line represents a haplotype and the frequency of the haplotype in this population is given at the end of the line. Haplotypes with a frequency below 2% are not included. Two SNPs are tagged and the SNP names are given in **C**.

Linkage disequilibrium was calculated between all the SNPs in the region between 33 and 44.9 Mb on SSC6. A large block of strong linkage disequilibrium in this area is observed. A part of this region, the area between 36.9 and 39.7 Mb is shown in Figure 4. All the 29 significant SNPs are present in only three major haplotypes in this population. Two copies of haplotype 1 has an average androstenone level of 2.13 μg/g and two copies of haplotypes 2 and 3 have an average level of 1.44 μg/g and 1.54 μg/g, respectively (Figure 4B). None of the remaining chromosomes show a comparable convincing cluster of closely linked SNPs associated with androstenone levels. Only isolated SNPs approach the significance threshold on SSC6 and SSC16 at 122 Mb and 105 Mb, respectively.

## Discussion

### Filtering of SNP data and statistical analyses

Quality control of the SNPs was based on the GenCall score, MAF and pedigree errors. Hardy-Weinberg equilibrium (HWE) was not considered relevant as a quality control tool as HWE is underpowered to detect genotyping errors [35] and only extreme sib pairs have been genotyped. GWA studies are particularly prone to spurious associations because ten thousands of associations are tested inflating the rate of false positives [36,37]. In this study, FDR was used to control for false-positive associations due to multiple testing. The genomic control approach was used to account for spurious association due to population stratification [30] and because the breeding line is a composite line derived from three different breeds. Correction for the inflation by division reduces the unadjusted p-value to adjusted levels and accounts for relatedness between the sib ships and possible population stratification. However, in this study the deviation from the chi-square distribution under the null-hypothesis (no association) was very low (λ_GC _= 1.06).

### QTL areas

**Figure 5:**
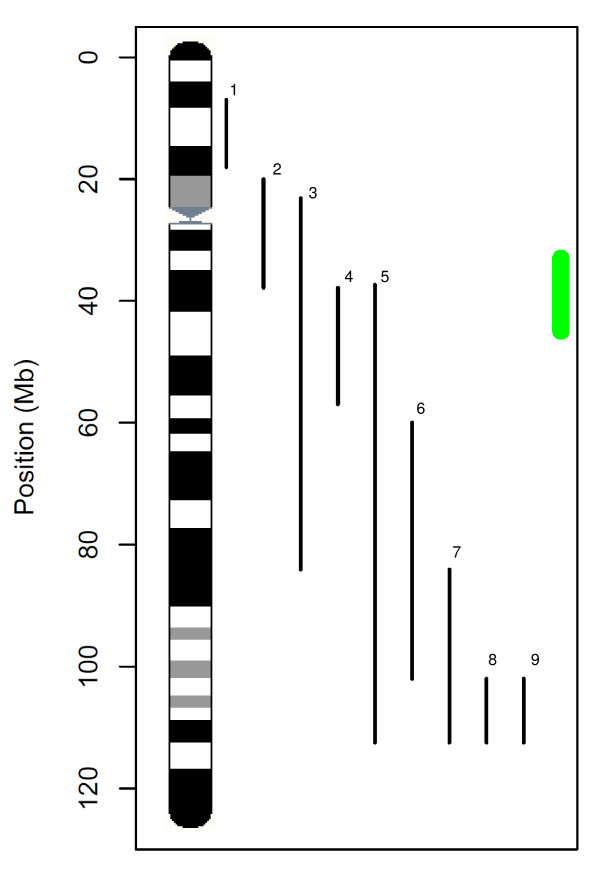
**Location of the QTL from PigQTLdb for boar taint traits on the physical map of *Sus scrofa *build9 SSC6**. The references and traits of the QTLs are given in Table 3. Positions in Mb were deduced from a BLAST alignment with the microsatellite markers. The green bar indicates the region found in this GWA study between 33 Mb and 45 Mb.

Mainly two chromosomes harbour highly significant associations with fat androstenone levels, a rather broad area of 10 Mb on SSC6, and three different regions on SSC1. For each region on SSC1 only 1-4 SNPs pass the significance level, whereas on SSC6 a total of 31 highly significant SNPs are detected. This is for the first time that an association with androstenone or related boar taint traits has been reported on SSC1. However, several studies have described QTL effects for traits related to boar taint on SSC6 (Figure 5). In an experimental F2 cross with Large White x Meishan, Lee et al. (2005) [17] reported a QTL for androstenone level in fat on SSC6 partially overlapping with the area identified here. In the same study, QTLs from a sensory panel for subjective pork flavour and boar flavour in lean meat were described that are also located nearby the area in our study. Finally, Szyda et al. (2003)[38] identified a QTL for smell intensity in a Duroc X Norwegian Landrace cross covering the area of interest. Considering the low resolution of these QTL studies, it is not possible to conclude whether they might be caused by the same genes segregating as in our study. The remaining boar taint QTL previously identified on SSC6 for smell intensity [39], subjective pork odour and skatole measurements [17,40] are located distal or proximal on SSC6. None of the other QTL studies investigating androstenone or sensory panel traits identified effects on SSC6 [19,24,39].

### Candidate Genes

For the two major areas of interest, genes potentially affecting steroid synthesis and metabolism of androstenone are listed in Table 2. The region on SSC 6, which is an extremely gene-dense area, shows several candidate genes located closely together between 37 - 38 Mb. Hydroxysteroid sulfotransferaseA1 (*SULT2A1*) maps to the homologous region in human and has not been annotated in the pig genome sequence. However, a BLAT search with porcine cDNA identifies 100% homology with two exons and including 3'untranslated sequence (pos.380-508, and 780-999). *SULT2A1 *catalyses the sulfoconjugation of 16-androstene-steroids in liver [41] and testis [42] and has been analysed earlier as a candidate gene for androstenone. Testicular activity of the enzyme was shown to be negatively correlated with fat androstenone levels in Yorkshire boars [43]. However, Moe et al. (2007) [21] found an increased expression of *SULT2A1 *mRNA in testis of Duroc and Landrace boars with high androstenone levels. In the same study, *SULT2B1 *expression was also increased in Landrace animals with high androstenone levels. *SULT2B1 *is located near *SULT2A1 *in human, but it has not been annotated in the pig genome sequence and cannot be located by BLAT alignment either. The overexpression of *SULT2B1 *does not explain the role of the sulfotransferases as inactivating enzymes [21]. *SULT2B1 *is selective for the sulfation of 3ß-hydroxysteroids, and Falany et al. (2006) [44] suggest a role in regulating the responsiveness of cells to adrenal androgens by reducing their conversion to more potent androgens and estrogens. In human, SULT2B1b is not expressed in the liver, however the different physiological functions of the two isoforms remain to be analyzed. Another conjugating enzyme also located in this area is *HSD17B14*. Differential expression of different hydroxysteroid dehydrogenases of the HSD17ß family (*HSD17B4, HSD17B11*) in the testis has been reported by Moe et al. (2007) [21]. *HSD17B4 *catalyzes the last step of androgen and estrogen synthesis. However, the function of *HSD17B14 *has only recently been investigated in human, and Lukacik et al. (2007) [45] suggest a role for the local inactivation of steroids in the nervous system and placenta. Northern blot analysis of human tissues shows that the gene is highly expressed in the liver, but not in testis. Adjacent, another new candidate gene, *LHB *is located, forming the ß-chain precursor of the luteinizing hormone (LH). At onset of puberty, LH is secreted by the pituitary gland and induces steroid synthesis in the Leydig cells of the testis. Interestingly, the gene encoding the α-chain of this glycoprotein hormone (CGA chorionic gonadotropin A) is located on SSC1 in the area of 58 Mb which also shows a significant effect in this study. No known or potentially interesting candidate genes could be pinpointed for the remaining two regions around 146 and 290 Mb on SSC1. Finally, the region on SSC6 extends to a second peak of SNPs nearly reaching the significance threshold around 33 Mb (additional file 2). Several cytochrome P450 genes of the *CYP2 *family are located there. This *CYP2ABFGST *cluster contains genes from multiple subfamilies [46]. In human, *CYP2A6*, *CYP2A7*, *CYP2B6*, *CYP2A13*, *CYP2F1 *and *CYP2S1 *cluster together. From these, aromatase (*CYP2A19*), which is the homolog of *CYP2A6 *in pigs, is known to catalyze the synthesis of estrogens from androgens. The pig expresses two isoforms in the testis. Moe et al. (2007)[21] have shown an upregulation of both isoforms in testis and liver of high-androstenone boars. Recently, Moe et al. (2009) [22] reported SNPs within candidate genes associated with androstenone levels in a commercial Duroc line. However, none of the candidate genes reported by Moe et al. (2009) [22] overlap with the major regions identified here.

**Table 2:** Candidate genes derived from porcine Ensembl build9.

Chromosome	Start position	End position	Porcine transcript	Gene
SSC1	58190063	58192283	ENSSSCT00000004751	Glycoprotein hormones, α chain
SSC6	33615478	33622941	ENSSSCT00000003325	*CYP2A19*
SSC6	33821587	33821766	ENSSSCG00000003001	*CYP2A6*
SSC6	37189463	37189682	ENSSSCT00000003463	Sulfotransferase
SSC6	37567155	37586075	ENSSSCT00000003479	*HSD17B14*
SSC6	37754569	37755346	ENSSSCT00000003498	*LHB*

**Table 3:** Overview of the identified QTL and flanking microsatellites on SSC6 for traits related to boar taint.

Nr.	Trait	Flanking markers	Reference
1	Subjective pork odor	SW1353 - SW1057	Lee et al., 2005 [17]
2	Subjective pork flavor in lean	SWR1130 (SW492) - SW782	Lee et al., 2005 [17]
3	Smell intensity	S0087 - S0003	Szyda et al., 2003 [40]
4	Androstenone, laboratory	SW782 - SW1823 (SW316)	Lee et al., 2005 [17]
5	Subjective boar flavor in lean	SW782 - SW322	Lee et al., 2005 [17]
6	Skatole, laboratory	S0059 (SW1473) - S0121 (S0299)	Varona et al., 2005 [42]
7	Smell intensity	S0003 - SW322	Grindflek et al., 2001 [41]
8	Skatole, sensory panel	S0121 (S0299) - SW322	Lee et al., 2005 [17]
9	Skatole, laboratory	S0121 (S0299) - SW322	Lee et al., 2005 [17]

When the flanking marker could not be placed on the physical map (*Sus scrofa *build9) then the nearest marker on the MARC map was used to estimate the physical position (marker name in brackets) of the marker.

Taken together, there is overwhelming evidence from previous QTL studies, candidate genes and differential expression that the region on SSC6 contains genetic elements affecting androstenone levels in boars. In order to disentangle the effects of the regions containing the *CYP450 *genes and the area around 37 Mb, a mixed-model analysis combining the effects of two SNPs (H3GA0052956 at 33.5 and MARC0049189 at 38.3 Mb) was performed. In this model the fraction of the phenotypic variance explained by both SNPs is 2.1% and 3.6% and together 5.7%. This means that both regions explain a part of the effect of the whole region but due to the high LD between the SNPs they capture the same variation individually (5.76%, additional file 1). Therefore, both areas remain relevant for the determination of androstenone levels in this population. This breed is a composite line which could explain this large extent of LD. More data from other unrelated lines or crossbred animals showing the same effect are needed to further reduce the region of interest.

### Effect size and application for breeding

Due to the skewed distribution of androstenone levels, even the use of a single marker would reduce the proportion of animals surpassing the threshold for consumer acceptance of 2 μg/g fat considerably. The difference between the two homozygous genotypes amounts to 15.6% (Figure 3). Sorting all offspring by the estimated androstenone effect of marker 50 and comparing the haplotypes of the 10 highest animals shows that all individuals are homozygous for the first haplotype shown in Figure 4. Furthermore, this haplotype is completely absent in the group of 10 animals with the lowest effects (data not shown). The 5 major SNPs (SNP nr. 1, 5, 6 on SSC1 and SNP nr. 15, 124 on SSC6) on SSC1 and 6 together explain 8.8% of the phenotypic variance, and considering a heritability of 64% [47] they account for 13.7% of the additive genetic variance.

A sustainable breeding scheme takes also into account the correlated effects on other production and reproduction traits. In general, the genetic correlations with growth, fatness and muscle depth are very low and favourable and therefore no serious negative effects on genetic progress due to selection against androstenone are to be expected [47]. Also, the positive genetic correlation with skatole would reduce skatole levels indirectly. However, the genetic correlation with fertility traits needs special attention. Male fertility data are not available on the animals in this study because they were slaughtered as commercial fatteners. Female fertility observations are only available on related animals and therefore estimates of genetic parameters have large standard errors (data not shown). A more extended study is underway to monitor the effects of selection against androstenone on male and female fertility. Furthermore, the effects in other lines that form part of the crossbreeding scheme to produce fattening pigs will be investigated.

## Conclusion

This study clearly shows the large increase in resolution of high-density SNP panels compared to earlier linkage studies using microsatellite markers. Several regions in the genome affect androstenone levels in fat in this commercial breeding line of pigs. The genome-wide significant SNPs detected on SSC1 and SSC6 show moderate to large effects explaining a fraction of the phenotypic variance of 2-6%. The candidate genes identified in these areas in the pig genome or via the comparative map in human include genes investigated in earlier reports. In addition, new genes from the pathways of the synthesis and metabolism of androstenone such as *LHA*, *LHB*, and *HSD17B14 *are detected. The rather large LD block seen in this population around 33-45 Mb on SSC6 prevents to disentangle the combined effects of these genes and to pinpoint more specifically the responsible genetic elements. Nevertheless, the most significant SNPs can already be used to accelerate genetic progress in breeding against androstenone in this sire line. However, genetic correlations with production traits and especially possible negative effects on fertility traits will deserve special attention.

## Authors' contributions

ND was involved in sample collection, design of selective genotyping, organization of the genotyping experiment, conducted statistical analyses, prepared figures and tables, and took part in writing the paper. EFK was involved in planning the project, statistical supervision and experimental set up. JWMM initiated the study, was involved in planning of the project and organization of the genotyping experiment. RPMAC was involved in DNA collection and the organization of the genotyping experiment. MAMG mapped all SNPs to porcine genome build9, identified human-porcine orthologous genes and was involved in the analysis and discussion of the comparative mapping data. HB was involved in the discussion and evaluation of statistical issues. BH coordinated the study, investigated the comparative map and selection of candidate genes, was involved in discussion on statistical issues, and drafted the paper. All authors have read and approved the final manuscript.

## Supplementary Material

Additional file 1 **Association results for corrected ln-androstenone**. If the DbSNP accession number was not available for a SNP, the forward and top genomic sequence of the SNP is given. Click here for file

Additional file 2 **Chromosome 6 close-up with the -log (Pval) from the PLINK analyses**. Above a -log p-value of 4.35 a SNP is considered significant. The start positions of porcine genes (n = 351) from EnSembl are plotted underneath as vertical grey bar based on the sequence of *Sus scrofa *build9. Vertical bars indicate the interval chosen for LD analysis in Figure 4. Click here for file

Additional file 3 **The annoted genes on SSC 6**. The start and stop position of the genes are given in bp and are based on *Sus Scrofa *build9. BLAT score and human gene name is also provided. Click here for file
