# Supplementary figures and images for: A genome-wide association study on androstenone levels in pigs reveals a cluster of candidate genes on chromosome 6
Source: BMC Genet. 2010 May 20;11:42. doi: 10.1186/1471-2156-11-42 (PMC2889844; doi:10.1186/1471-2156-11-42)

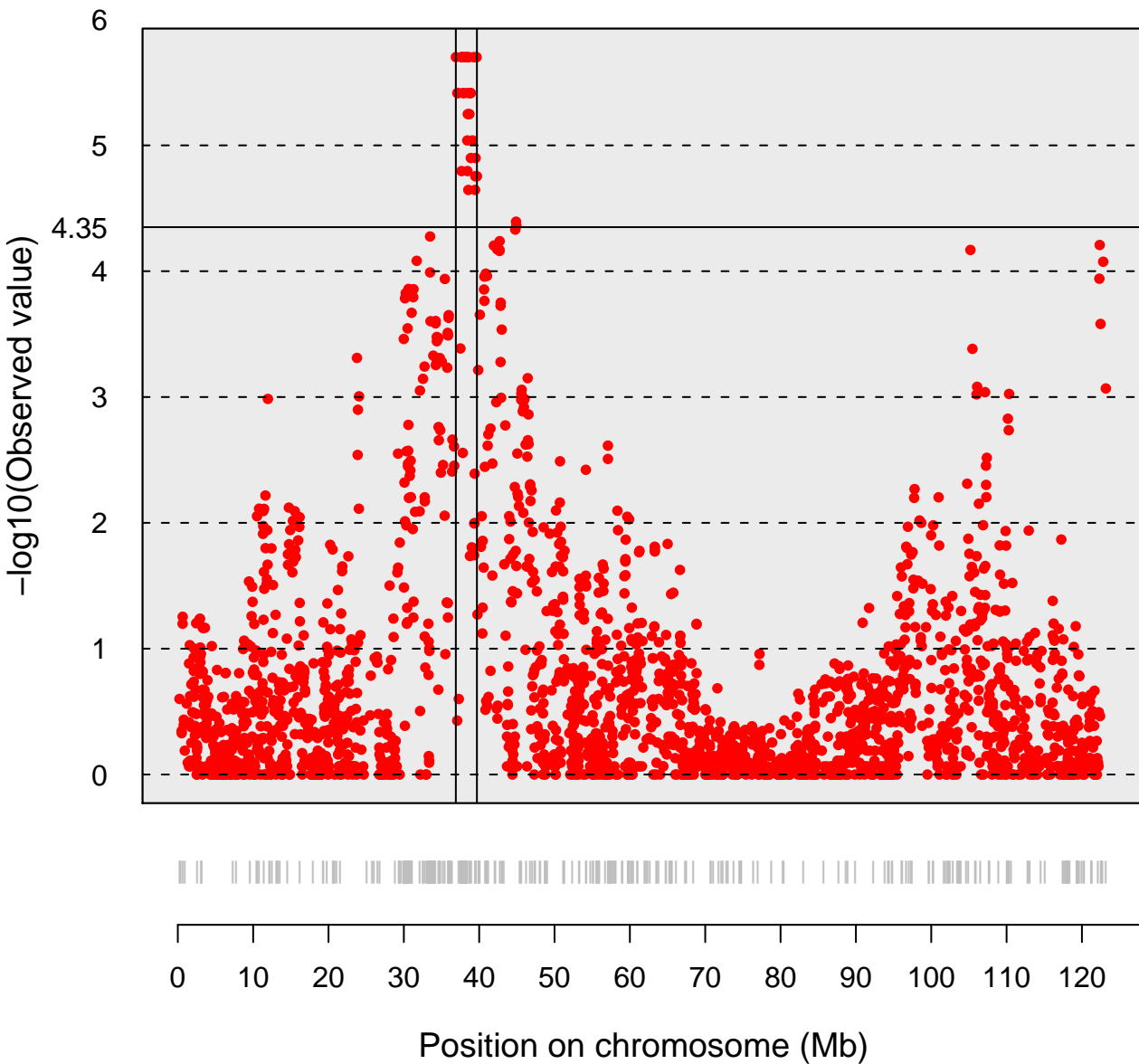

Supplement: Additional file 2 — Chromosome 6 close-up with the -log (Pval) from the PLINK analyses. Above a -log p-value of 4.35 a SNP is considered significant. The start positions of porcine genes (n = 351) from EnSembl are plotted underneath as vertical grey bar based on the sequence of Sus scrofa build9. Vertical bars indicate the interval chosen for LD analysis in Figure 4. [file 1471-2156-11-42-S2.PDF]
